# Achieving Digital Health Equity by Personalizing the Patient Experience

**DOI:** 10.1089/tmr.2023.0018

**Published:** 2023-06-27

**Authors:** Priya Bathija, Elizabeth A. Krupinski, Jorge A. Rodriguez, Tara Sklar

**Affiliations:** ^1^ Nyoo Health, Chicago, Illinois, USA.; ^2^ Department of Radiology & Imaging Sciences, Emory University, Atlanta, Georgia, USA.; ^3^ Brigham & Women's Hospital, Boston, Massachusetts, USA.; ^4^ University of Arizona, Tucson, Arizona, USA.

**Keywords:** virtual care, digital health inequities, telemedicine, strategies, solutions

## Abstract

**Background:**

COVID saw a significant increase in the use of virtual care, supporting its utility and its benefits. It also revealed that unfortunately there are limitations and gaps we still need to address, including inequitable access to digitally enabled health care tools.

**Methods:**

On November 8, 2022, the Mass General Brigham held the Third Annual Virtual Care Symposium: Demystifying Clinical Appropriateness in Virtual Care and What's Ahead for Pay Parity. One panel addressed digital health equity and key points are summarized here.

**Results:**

Four experts discussed the key domains of digital equity and inclusion in the session titled “Achieving Digital Health Equity: Is it a One-Size-Fits-All Approach or Personalized Patient Experience?” These included lessons from strategies and tactics being used by hospitals and health systems to address digital equity issues; and opportunities to achieve digital health equity for specific populations (e.g., Medicaid).

**Conclusions:**

Understanding the drivers of digital health disparities can help organizations and health care systems develop and test strategies to reduce them and improve access to quality health care through digitally enabled technologies and delivery channels.

## Introduction

COVID saw a significant increase around the world in the use of virtual care, supporting its utility and its benefits.^1,2^ It revealed a lot about what is feasible and where it could be of help. It also revealed that although virtual care is appropriate for many clinical specialties and patients, unfortunately there are limitations and gaps we still need to address.^3,4^

Some of these gaps are unique to certain countries (e.g., licensure and other legal requirements, reimbursement), but some of them are more widespread. One of the biggest gaps that became quite evident was inequitable access to digitally enabled tools and virtual care opportunities.^3,4^ There are many ways to address these gaps as no single solution will be relevant to every patient and health care institution; thus, the intent of this article is to provide some key examples, data, and lessons learned as a starting point for promoting change.

First, we need to understand the key domains of digital health inequity. Second, we can learn important lessons from the strategies and tactics being used by hospitals and health systems to address digital health inequities. Finally, we can examine the gaps and opportunities to achieve digital health equity for specific populations such as those eligible for Medicaid or government assistance programs.

Although the strategies and solutions discussed here are based primarily on experiences in the United States, they should be readily generalizable to other populations and circumstances around the world.

## Key Domains of Digital Health Equity and Inclusion

This section will cover three core themes: how to define digital health equity, why are we talking about this now, and how do we go about achieving digital health equity. Digital health equity can be thought of as everyone having a fair and just opportunity to engage with and benefit from digital enabled health tools.

This is borrowed from a medical-level health equity definition^5^ that everyone should have a fair and just opportunity to benefit from medical care. In our discussion, we are referring to the patient facing digitally enabled health tools such as mobile health apps, patient portals, remote monitoring, telehealth, and texting solutions.

So why are we talking about this now? As noted, there was a seismic increase in virtual care use over the past 2 to 3 years. In addition, during vaccine deployment, health systems frequently relied on online-based scheduling tools that brought to the forefront that certain parts of the population did not have access to the internet. Unfortunately, these were often the populations that were disproportionately affected by COVID.

Further, something less discussed, is the enactment of the 21st Century Cures Act,^6^ which put into place a law that empowered patients to have easier access to their health care data. Of course, that requires going online. If you think about HITECH Act^7^ and the development of patient portals, for example, the early literature highlights substantial disparities in portal use and access, with low rates among marginalized populations.^8,9^ So, it is not surprising that these disparities became starkly visible during the pandemic as demonstrated by the significant disparities in the use of video visits by marginalized populations.

When thinking about digital health equity there are five key domains: technology access, technology infrastructure, and health literacy (which incorporates English proficiency), implementation, policy, and standard of care. Technology access is the classic digital divide—those who have access to the internet and those who do not. However, subsequent work has highlighted that those digital inequities are multifactorial.

For example, broadband infrastructure (who has wired internet lines or Wi-Fi signal available in their home) and broadband affordability (are you able to afford it and the necessary devices) are additional factors driving digital inequities. These access gaps represent an opportunity for health care systems to serve as a touch point to determine whether we must screen for these things and then refer patients to the appropriate resources.

Structural barriers to digital equity, like digital redlining, are increasingly being recognized as critical to digital equity. Digital red lining or digital discrimination refers to a practice by internet service providers (ISPs) in which they deploy internet services in ways that disadvantage certain groups. For example, in some areas, ISPs deploy internet access at varied speeds and quality, but charge customers the same price. Customers living in underserved areas often pay the same amount for slower speeds.

Digital literacy or the use of digital tools presents key gaps and opportunities to achieve digital equity. Digital literacy is the “ability to use information and communication technologies to find, evaluate, create, and communicate information, requiring both cognitive and technical skills.”^10^ The design and development of the platform can affect a patient's ability to use digital tools.

For example, apps that are not designed for patients from underserved populations can limited their utility. Sarkar et al. found that patients from safety net clinics had difficulty in completing data entry and data retrieval tasks on chronic disease mobile apps.^11^ Even when the technology is designed optimally, many patients may still need some extra support and help to get connected and feel comfortable.

One strategy is the increasing use of digital health navigators to help people on-board or use these technologies as new members of the health care team. Prior work has piloted digital health navigators in primary care clinics. In the pilot, the navigator reached out to about 400 patients who were not active on the patient portal and supported patients in enrolling in the portal.

The team was able to contact most of them and enrolled about a third. Of those enrolled, about 80% logged into the portal again so this one-on-one support really made a difference in connecting patients with this new tool and making it part of something to use over the long term. Finally, it is critical to consider that digital literacy is not unique to health care. Collaboration with experts from adult education and youth education can impact a patient's life beyond health care, including in civic engagement, access to social benefit programs, public assistance, and workforce development.

From an implementation standpoint there are four things to highlight. First, we need to involve patients and families in the development and implementation of digitally enabled tools for equity, so they can help guide the digital experience. Patient preference can shape how digitally enabled tools are implemented in health care, and we must build systems that provide both digital and non-digital options. Second, the equitable implementation of digitally enabled tools requires monitoring use across patient demographics.

Then, organizations can create venues (e.g., newsletters, blogs, white papers) to share the data to those in leadership to highlight gaps and strategize on what to do to address them. Third, workflows can facilitate or hinder the success of digitally enabled tools, especially among underserved populations.

For example, workflows that include interpreters as part of a virtual care visit can ensure the use of digitally enabled tools among patients with limited English proficiency. Fourth, patient privacy, especially when sharing data remotely, must be maintained and patients educated on what security measures are in place, especially among communities that already have mistrust with the health care system.^12^

In terms of policy, the Infrastructure Investment and Jobs Act that was passed in 2022 includes a section on digital equity. It provides policy and funding to gaps in technology access and literacy. It makes digital equity a public focus, which has implications for the health care sector.^13^ For example, the Affordable Connectivity Program provides an internet and device subsidy for eligible patients who have these digital needs.

Further, it includes funding for digital literacy programs. This policy can serve as the foundation for health care organizations to partner with community organizations and develop community-wide digital equity programs. From a health care organization standpoint, being able to refer patients to these programs is one big opportunity for health care organizations.

Finally, we need to view digitally enabled tools as the standard of care available to all patients. Data suggest that only certain patients are offered these tools. In one study, Black and Hispanic patients were offered patient portals at significantly lower rates than White individuals.^14^ Clinicians and providers can play a significant role in promoting digital equity in delivering care. Conversations need to occur to make sure we do not act on implicit bias about who is going to use technology and who is not. The goal is digital health equity but only to the extent that it gets us to the ultimate goal of total health equity.

## Strategies and Tactics Being Used by Hospitals and Health Systems to Address Digital Equity Issues

Digital health solutions have become essential tools for health care providers as they deliver care. Therefore, making sure patients can access and understand mobile health apps, patient portals and virtual care must be addressed at both the system and patient levels.

At a system level, health care providers and technology companies can commit to designing and implementing strategies in a way that all patients can access and understand. In addition, they can commit to including digital health equity as part of the conversation, not only in the design phase but also in the implementation and evaluation phases.

For example, CommonSpirit (Chicago, IL) has embedded health equity and digital health equity into their strategy. They include questions related to technology access and digital health literacy in the process designed to evaluate digital solutions. They then go beyond this to evaluate the use of technologies.

Another system-level approach is to create a process that engages diverse patient voices. That can include partnering with community-based organizations that know the needs of patients and communities. It can also include creating avenues for under-represented patients, communities, and providers to give feedback on whether digital solutions are accessible and understandable by real patients and communities.

For example, hospitals could engage moms with low health literacy to understand their communication preferences and tailor services to meet their needs and build their trust during this important time of their life.

Another system-level solution is tracking patient engagement with digital tools to understand the patients and populations that are using these tools. CommonSpirit has implemented Docent Health, a program to provide culturally competent navigators to pregnant moms. These navigators are embedded in the communities that they are serving. CommonSpirit analyzes the data around the use of this program, stratifying it by race, ethnicity, and social vulnerability to determine which communities are engaging in Docent Health.

This analysis allowed CommonSpirit to not only know who is and is not using the solution, but it also allowed the team to learn that for some Spanish-speaking populations in California providing a Spanish-speaking navigator was not enough. Those patients needed a navigator who spoke the right dialect of Spanish for their specific community.

The last system-level strategy is to advocate for policies that allow patients to access digital health solutions.

For example, the American Hospital Association^15^ is currently advocating for investments in infrastructure, including broadband access as well as increased federal funding, coverage, and reimbursement for the expanded use of virtual care and other technologies.

Patient-driven strategies are also important drivers for digital health equity. While there are federal and state laws that must be considered, one way to improve digital health access for individuals is to provide patients and physicians with technology (including hardware and software).^16^

For example, the University of Mississippi Medical Center launched a pilot program network in diabetes virtual care. It provided patients in the program with tablet computers at no cost and they were then able to take and report their own vital signs daily, which led to improved patient outcomes, increased medication management, and patients were more willing to participate in virtual care visits.^17^

Health care providers can also proactively work with technology companies to select solutions that minimize barriers to access. When Boston Medical Center was looking for a way to provide remote patient monitoring to address postpartum hypertension, it considered a variety of different solutions.

The team at Boston Medical Center recognized that most of their patients had access to smartphones, but there was a divide in patients' ability to connect. Some patients did not have consistent access to Wi-Fi or did not have a data plan that could be used to support a video framework. Ultimately, the team selected Rimidi (Atlanta, GA), a solution that uses the local cellular network, making it accessible to anyone with a smartphone.

Health care providers and technology companies also may need to acknowledge that video-based digital solutions are not always the best path forward. One popular alternative is using asynchronous digital technologies such as SMS text messaging to provide information and an access point for patients to connect with health care providers. Research indicates that 90% of text messages are read within 90 sec of when they are received.^18^

As a result, texting can be a powerful way to reach patients with the right message at the right time. It can also be done in different languages, is relatively cost-effective, and can triage patient needs, provide timely information, and improve communication and engagement with patients.

Another strategy is to develop digital solutions that are linguistically and culturally sensitive and inclusive.

Providence (Renton, WA) uses Wildflower (San Francisco, CA), which provides information and resources to patients from pregnancy through delivery. The individuals who designed this solution realized quickly that they needed more than mere translation from English to Spanish; they needed to achieve localization to adequately meet the needs of their Spanish-speaking population.

At its core, translation transforms text, whereas localization (i.e., the process of adapting and transforming content so it resonates in another country or locale) transforms the entire product or content from one language to another (e.g., using metric vs imperial for measures, proper currency, use of emojis).

They needed to evaluate the functionality of the app and ask the question, how would a Spanish-speaking individual approach this app? As a result, they created a solution that considered individuals' cultures, as well as how, when, and where they would be using the app.

These solutions can also be tailored to meet the needs of those with lower digital literacy. For example, by using more videos, images, emojis, and symbols, providers can ensure that patients, regardless of their literacy level, are able to access and understand the information. In addition, those with low digital literacy may be afraid to use technology or trust their providers.

To address these concerns, health care providers can offer training to support their patients throughout the process. Similar to the digital health navigator referenced earlier, Ochsner Health (New Orleans, LA) launched the “O Bar” concept many years ago. The O Bar, located within Ochsner's Center for Primary Care and Wellness, carries physician-recommended digital products and is staffed by a full-time technology specialist that can help patients choose the right tool and help set up, guide, and support these individuals as they use those tools.

Nemours Children's Hospital (Wilmington, DE) redeployed staff as digital health navigators to help patients and their families complete digital forms, troubleshoot connectivity issues, and better engage in virtual care visits.

The last strategy is that organizations can develop workflows that better allow clinical teams to engage patients in these tools. Froedtert (Milwaukee, WI) and the Medical College of Wisconsin (Milwaukee, WI) implemented Babyscripts (Washington, DC), a platform designed to connect expectant mothers with doctors and resources to improve perinatal outcomes. Expectant mothers are invited to join Babyscripts after it is determined that they are pregnant.

However, early in the implementation, Froedtert realized that certain communities and populations were not engaging with the app. They created a process where their team proactively reaches out to individuals who do not accept the initial invitation and encourages them to join and use the Babyscripts tool, and this has a positive impact on enrollment.

## Digital Equity and Inclusion in Medicaid Beneficiaries

Medicaid provides health benefits and other services for low-income individuals. There are about 76 million Americans on Medicaid, and dual-eligible patients are those older adults on Medicare who are also low income, accounting for over 12 million Americans. The Government Accountability Office recently published an analysis of five state experiences and how they were using virtual care access to care for Medicaid populations.^19^

They examined virtual care utilization among Medicaid beneficiaries pre-pandemic (before March 2020) and then what happened through February 2021, where there was an incredible 15-fold increase across the 5 states. They choose states representing a broad swath of population in terms of percent in rural areas, having access to broadband, and variations among demographics such as age, income, and education.

Right before the pandemic, about 11% of Medicaid beneficiaries (e.g., in Arizona) were accessing virtual care for one or more of their health care services. In the year the pandemic started (March 2020 to February 2021), that shot up to 43.8. Thinking about this increase in virtual care use with regards to a very specific very vulnerable population and how they were able to access virtual care changes the picture. Medicaid supports those who qualify through means, so their income thresholds are below a certain amount.

But there are also subgroups who have disabilities as well as being low income and those who are older, and they have specific health care needs, including behavior health for many with disabilities. Given the need for behavioral health services combined with limited staffing, the majority of states now provide Medicare coverage for audio-only behavioral health services.^20^

For older adults, long-term care is a big issue and Medicaid is the primary source of coverage for long-term services and supports (LTSS) in America. Thus, when we think about digital health tools and access to health care via virtual care, we need to consider how it would apply in these settings.

Currently, every state has a waiver for home- and community-based services where, in the case of older Medicaid beneficiaries and those with disabilities and chronic illnesses, they have the option to go into a nursing home to receive care or they can live independently and receive LTSS in their home to assist them with their daily needs.

The problem is that the demand to age in place far outweighs the supply. Across the United States, Medicaid-eligible older adults are waiting an average of 3 years across with over 820,000 Americans waiting to receive care in the home instead of going to an institutional setting.^21^

This not only affects Medicaid-eligible older adults but also has a real impact for our whole society in terms of how we are caring for an older population. Digital health tools and the ability to have access to care via virtual care in the home supports individuals aging in place, which is a goal that many Americans share.

This is happening incrementally. During the pandemic, Appendix K (from Medicaid.gov it is part of the Emergency Preparedness and Response for Home and Community Based (HCBS) 1915(c) Waivers—a standalone appendix that may be utilized by states during emergency situations to request amendment to approved 1915(c) waivers.

It includes actions that states can take under the existing Section 1915(c) home and community-based waiver authority to respond to an emergency) was added to home- and community-based waivers that many states took advantage of trying to digitize home- and community-based services to allow for more digitally enabled health tools in the home, and allow virtual care visits as opposed to in-person care.^22^

That could be a way going forward, as states are increasingly considering options available in Medicaid waivers to provide a greater number of seniors with LTSS in the home and off the waiting lists.

If you have very limited means, it makes a big difference in terms of how health care can be reimbursed. Payment parity means the same amount of reimbursement for in-person care as for virtual care, whereas coverage parity means that the service is simply covered. If it is not covered at all, how would an individual with very limited means receive the care they need and would prefer?

States were doing different things before the pandemic started and then changed during the public health emergency to allow access to virtual care for Medicaid beneficiaries from their homes. Notably, behavioral health has greatly benefited from virtual care options and its use was high during the pandemic and has remained high.

Other specialty services experienced a huge jump, particularly dental, occupational health, and long-term supports and services. Many states followed Medicare's approach to expanding coverage for care delivered via virtual care during the pandemic, and modified regulations for their Medicaid populations in terms of widening the numbers of providers and types of services available to them.

Further, data on virtual care utilization among Medicaid beneficiaries were further broken out by accessing services with video/audio care as well as audio-only. Some data broke use down by race and ethnicity, income, and education levels.^23^ For the most part, white individuals were much more likely to access virtual care via video/audio; whereas those who were of a Latino background were much more likely to access virtual care via audio-only. Thus, if we limit access to care in ways to be only video/audio, it will impact certain population groups within this already very vulnerable Medicaid population.

There are other ways to enhance access to care outside of state legislation and governor executive action, and that is what individual Medicaid departments are doing in certain states. California's Department of Health Care Services, which operates the state Medicaid program, released a list of virtual care modifications that include payment parity for audio-only care as an example of how they were trying to ensure care for their vulnerable population group; and Ohio's Department of Medicaid permanently expanded coverage for audio-only and authorized a board range of providers and services eligible to deliver care virtually to their Medicaid-eligible population.

Other states craft their legislation around virtual care definitions. Arizona has a detailed approach to how virtual care is defined in AZ HB 2454 with a very specific audio-only carve-out in which only in situations due to technology or other infrastructure limits would it be considered appropriate. This audio-only carve out has real implications for access to care in Arizona.

The state legislature created an advisory council that initially recommended to reduce the number of codes that would be eligible for audio-only by nearly two-thirds but has since then amended their recommendation to align audio-only coverage with Medicare.^24^ In contrast, Colorado's definition is quite broad—any type of care delivered at a distance without an audio-only carve-out in CO HB 1190. This broad definition allows for emerging technologies to be used.

Thus, if we require video/audio services or in-person visits this will impact access to care, especially those in vulnerable patient-population groups who disproportionately access care via audio-only. Interestingly, the Federal Trade Commission says it is the standard of care that matters, not how it is delivered.

A final example is what has been happening across the country when it comes to rural populations trying to access palliative care. Patients must make really tough decisions between being able to remain in their home and have their pain managed or going to more urban or tertiary centers to receive palliative care.

This has been shown to have a significant impact on patients being able to access palliative care in their home and is a huge equity issue just allowing patients to have that decision.^25,26^ A large randomized trial of telepalliative care is underway and will provide significant insight into the role of digitally-enabled care delivery for this population (NCT01038271).

## Summary

What can the individual practitioner do to utilize virtual care through the lens of health disparities and health equity? Simply talking to the patient is a great starting point—what is the pain point the patient is feeling and is there a technology that can address it, and if not are there other options?

Sometimes we try to think of technology very broadly, but for the individual patient we need to tie it to a framework with health equity in mind moving toward structural and social determinants of health. The health care community need to identify through these conversations what the challenges are how do we bridge them.

We need to talk with patients about what they want. Some dislike using technology and want in-person visits all the time as they enjoy the communication and conversation. Some on the other hand are very tech savvy and want everything done through an app or portal.

We need to ask these questions and not make assumptions about what someone would prefer just because they are old or young, male or female, etc. However, once the questions are asked, we need to acknowledge and address the fact that many providers do not have the resources needed to support the preference that an individual may have.

We need to better embed resources in the system. At the first point of engagement, we can ask “How would you prefer to have your visit conducted?” then let people choose between text, email, audio only, visual regardless of their payor—Medicare, Medicaid, private, out of pocket, and treat patients equally across the board. We also must educate patients as to what these terms (e.g., telemedicine, eHealth, virtual health).

There are many stories about how patients picked the virtual option then at the scheduled time do not realize what they picked and that it meant needing a computer and internet to connect. Even though people can go through a checklist and pick options, it is not just a matter of do they have the access, but do they understand what these options are. Without these basic one-on-one conversations about what options are available, what they entail and whether resources exist, digital health equity will not be attained.

## Abbreviations Used

ISPs: internet service providers
LTSS: long term services and supports
